# Cloning: A Review on Bioethics, Legal, Jurisprudence and Regenerative Issues in Iran

**Published:** 2016-09

**Authors:** Seyedeh Leila Nabavizadeh, Davood Mehrabani, Zabihallah Vahedi, Farzad Manafi

**Affiliations:** 1 Legal Office, Vice Chancellor of Management Development Resource Planning, Shiraz University of Medical Sciences, Shiraz, Iran;; 2 Stem Cell and Transgenic Technology Research Center, Shiraz University of Medical Sciences, Shiraz, Iran;; 3 College of Law, School of Art, Shahed University, Tehran, Iran; 4 Iran University of Medical Sciences, Tehran, Iran

**Keywords:** Cloning, Bioethics, Legal, Jurisprudence, Regenerative medicine, Iran

## Abstract

In recent years, the cloning technology has remarkably developed in Iran, but unfortunately, the required legal framework has not been created to support and protect such developments yet. This legal gap may lead to abuse of scientific researches to obtain illegal benefits and to undermine the intellectual property rights of scientists and researchers. Thus to prevent such consequences, the attempts should be made to create an appropriate legal-ethical system and an approved comprehensive law. In this review we concluded that the right method is guiding and controlling the cloning technology and banning the technique is not always fruitful. Of course, it should be taken into accounts that all are possible if the religion orders human cloning in the view of jurisprudence and is considered as permission. In other words, although the religious order on human cloning can be an absolute permission based on the strong principle of permission, it is not unlikely that in the future, corruption is proved to be real for them, Jurists rule it as secondary sanctity and even as primary one. If it is proved, the phenomenon is considered as example of required affairs based on creation of ethical, social and medical disorders, religious and ethical rulings cannot be as permission for it, and it seems that it is a point that only one case can be a response to it and it needs nothing but time.

## INTRODUCTION

The word “cloning” is referred as “making an identical copy” which has a Greek origin of “Asexual replication of an organism”. Cloning has been used in various fields of biology while the DNA molecule of cells with genetically identical structure is known as a clone. Honey bees propagate by cloning as the queen bee mates once during her life and the eggs propagate in the queen up to thousands of eggs that are further hatched into bees.^1^ Although Joshua Lederberg advocated cloning and genetic engineering as a subject of speculation in 20th century, scientists and several authorities started to take the prospect seriously in the mid-1960s.^2^ James D. Watson was the person who publicized the potential and the perils of cloning in 1971.^3^ With the cloning of a sheep by somatic cell nuclear transfer (SCNT) called Dolly, the idea of cloning of human has become a hot debate subject.^4^ Advanced Cell Technology in November 1998 by using SCNT created the first hybrid human clone. A nucleus was taken from a man’s leg cell and was introduced into a cow’s egg while its nucleus was removed. The hybrid cell was cultured, and developed into an embryo and after 12 days, the embryo was destroyed.^5^

In 2004 and 2005, pluripotent, embryonic stem cells were successfully harvested from a cloned human blastocyst using SCNT and eleven different patent-specific stem cell lines were created as the first breakthrough in cloning of human.^6^ In January 2008, the first five mature human embryos using SCNT were created while each embryo was created by taking a nucleus from a skin cell and inserting it into a human egg from which the nucleus was removed. The embryos could be developed only to the blastocyst stage, and were destroyed later. The “holy grail” that was useful for therapeutic or reproductive cloning was used to generate embryonic stem cell lines.^7^^-^^9^

In 2011, the New York Stem Cell Foundation could generate embyronic stem cell lines, resulting in triploid cells, which were not useful for cloning.^10^^-^^12^ In 2013, embryonic stem cells were created using SCNT. Four embryonic stem cell lines were derived human fetal somatic cells using oocytes from the same donor, ensuring that all mitochondrial DNA inherited was similar.^10^ Advanced Cell Technology reported replication of Mitalipov’s results and showed the effectiveness by cloning adult cells using SCNT.^4^^,^^13^ So cloning has attracted attention of physicians, medicolegal specialists, and other scientific circles as it has opened a new window to the human with its therapeutic advantages but with some concerns too.^14^

The UNESCO declaration on human genome, the human rights of 1997 and the European Convention on Human Rights and Biomedicine (Strasburg) proposed concerns with this scientific phenomenon and the experimentation on human.^15^ After the emerge of human cloning, the legislature passed laws regarding requirements, structures, resources and the evolving capacities of civil rights and the future legal researches, resulting in irreparable consequences; especially, for concerns about human rights and criminal law in the third millennium.^16^

In recent years, the cloning technology has remarkably developed Iran, but unfortunately, the required legal framework has not been created to support and protect such developments yet. This legal gap may lead to abuse of scientific researches to obtain illegal benefits and to undermine the intellectual property rights of scientists and researchers. Thus to prevent such consequences, the attempts should be made to create an appropriate legal-ethical system and an approved comprehensive law.^14^

Law and ethics are basic and fundamental concepts in this area and according to 4^th^ principle of constitution law stating that all laws should be in the framework of Islamic regulations and as there is not any specific law related to human cloning in the country, we should refer to accredited judicial decree or ethics. On the other hand, based on principle 177, constitution law, it is an unchangeable principle and it has been constant after all reviews. Although in bioethical and jurisdictional point of view, the status of reproductive and therapeutic cloning is analyzable, and sanction is the legal status of the matter to be required and of great importance.^17^

Therefore, legislators should take actions toward criminalization of the issue with respect to principle of legality of crime and punishment. One of the primary and certain principle of criminal law is the principle of legality of crimes and punishments; that is, briefly: first, no action is a crime unless it is already known and attributed as a crime by the legislator; second, no punishment is possible to be ruled unless it is already passed to be executed for the crime by the legislator.^17^ The major objective of this review is the legal analysis of the subject. However, the related bioethical and jurisprudential aspects will be discussed. 

## APPLICATIONS OF CLONING

Work on cloning techniques has advanced our knowledge on developmental biology, especially early human development. Basic understanding on signal transduction together with genetic manipulation within the early human embryo has the potential to respond to many developmental diseases and defects requiring aesthetic and regenerative medicine to enter the field.^18^ Cells created by SCNT are beneficial in research of the causes of diseases, and as model systems for drug discovery^19^^,^^20^ Cells produced with SCNT could eventually be used in cell transplantation,^21 ^or for creation of organs in transplantation, called regenerative medicine. Stem cell therapy is cell transplantation in treatment or prevention of a disease or condition.^22 ^Bone marrow transplantation is a widely used form of stem cell therapy.^23 ^The potential use of stem cell therapy in treatment of several diseases is underway.^24^^,^^25 ^Regenerative medicine would allow autologous transplantation of stem cells, and removes the risk of organ transplant rejection by the recipient.^26 ^For instance, in liver diseases, a new liver may be grown using the same genetic material and transplanted to remove the damaged liver.^27 ^Human pluripotent stem cells have been promised as a reliable source to generate human neurons, with the potential for regenerative medicine in brain and neural damages.^28^

## HISTORY OF CLONING

Cloning is the outcome of the hard works on use of genetic engineering in animal breeding, treatment of hereditary diseases in human and replicating organisms.^16^ In 1901, transfer of nucleus of a salamander embryonic cell to a enucleated cell was successfully undertaken. During 1940-1950, scientists could clone embryos in mammals. In 1956, Spemann’s hypothesis was proved and in 1962, mature frog was produced by transferring nucleus of intestinal cells of tadpoles into the eggs while their nucleus were removed.^29^

Sheep cloning from embryonic cells was performed in 1984. In 1994, bovine cloning was conducted from embryonic cells of another cow. In 1996, first cloned animal called Dolly was produced in Scotland using mature cells of mammary glands of a mature sheep. The importance of Dolly was for its production from differentiated cells of mammary glands while the previous cloned animals were produced from embryonic cells. The birth of Dolly led to undermining the impossibility of simulation by differentiated and specific cells. In the late 2000, scientists cloned 8 species of mammals. In 2003, the first cloned mule was produced by the American scientist. In 2005, the first cloning of a dog called Snoopy was carried out. In 2006, the Iranian scientists succeeded to clone a few sheep among the Middle East countries.^29^

Bonyana was the first cloned calf in Iran. The birth of this calf was the outcome of a series of researches from 2003 to produce various livestock by IVF. Cloning and genetic engineering lead to the birth of Royana, the cloned sheep and Hanna, the cloned goat.^30^ Tamina was the second cloned calf in Iran and it was cloned from the cell origin similar to Bonyana, the first cloned calf. This calf was born with the weight of 70 kg by Caesarian operation in Foka Animal Breeding Complex affiliated to Social Security Organization after the 280-day pregnancy period but after a few hours died due to an acute brucellosis, while Tamina also showed the signs and symptoms of some anatomic disorders at birth.^30^

## HUMAN CLONING

*Reproductive Cloning*

Reproductive cloning is the process where the asexual cells are transferred to an egg while its DNA has been removed and after the development of an embryo, it is placed into the recipient uterus. This process can result in production of a human while the cloned individual would totally be identical to the genetic donor.^15^

*Therapeutic Cloning*

The therapeutic cloning also known as embryonic cloning is actually used to produce human embryos for research purposes. The objective of this type of cloning is not the production of a cloned human but the culture of cells is used in human researches and for treatment purposes in regenerative medicine. These cells are very important for biomechanics researchers because they can be used to produce any types of cells of human body. These cells are extracted from embryo after 4 days of cell division. The process of extraction ruins the embryo and this issue creates a lot of ethical concerns. The researchers hope to replace the cloned cells for the cells destroyed by diseases such as Alzheimer’s, cancer, etc.^31^

*Advantages of Cloning*

The cloning technology may have positive and negative effects with advantages as well as disadvantages and even can be with fatal effects. The most important advantages of cloning can be (i) Replicating and propagating plants and animals, (ii) Recreating and replicating extinct or going to extinct animals, (iii) Propagating genes and saving newborns from hereditary diseases, (iv) Helping to discover treatment methods of infertility, (v) Dividing the developed embryo into several cloned embryos so that in case of probable incidents happening to one of them, the other clone can replace it, (vi) Using it to reproduce the ambulated limbs and replicating them to culture and replace the destroyed organs such as liver, heart. One of the advantages can be that the cloned limbs have full genetic adaptation with the recipient individual who is the donor of the stem cells, (vii) Helping to control population regarding shortages of male or female sex due to incidents such as war and earthquake, and (viii) Helping to reduce sorrows and pains of people suffering from the death and absence of their loved ones by cloning them.^32^

*Disadvantages of Cloning*

Because this technology is new and its outcome is not public and common yet, the damages and losses are sometimes resulted as internal damages by nature of the operation and the process of cloning. Sometimes, there are external damages imposed on the cloned society or individual after the cloning operation. Internal damages may be (i) The cloned living organism may encounter genetic problems and complications in long term, (ii) The more the cloned people are in the society, the more their extinction probability will be; because there are about one million four hundred thousand nucleotides in the body of every human and this remarkable variety is the origin of human generation survival; while the decrease in the genetic variety of individuals in a society, which is the result of cloning– highly increase the probability of their death by a special virus or a pathogen, (iii) 99% of attempts to clone human may result in creation of monsters, (iv) Biological disorders such as cancer, (iv) Premature aging: Dolly, the sheep, aged soon after cloning and the cloned baby will age at birth; because if the genetic donor is fifty-year-old, the new born will be a fifty-year-old one, thus, it will be suffering from premature aging like Dolly.^32^

External damages can be (i) Belief damages, (ii) Human moral damages, (iii) Cloning propounds a way to stop family establishment and perseverance against the related difficulties and it leads to satisfying sexual instinct and contenting oneself with cloning to have a child, (iv) Cloning is against divine nature. The nature of human and other living things is based on marriage tradition and the Holy Quran frequently emphasized on the creation of human based on the marriage tradition, but cloning is independent of either one of the couples. Besides, marriage has advantages and useful effects such as comfort, friendship, kindness and love in addition to reproduction and propagation of generation and such emotions ruins in cloning.^32^

(v) Cloning can result into harmful side effects for the individual like other unnatural methods in medicine. The use of powder milk for breast milk, Caesarian operation for natural delivery, etc. has brought a lot of problems for the individuals and they are not recommended unless required. Cloning will have the same side effects and problems and because there is not a necessity for its operation, and bearing such health and social damages are not scientifically justifiable. A healthy body can affect mental health as proper nutrition does on physical health too. Therefore, regarding children nutrition, it can indirectly be useful to improve mental and spiritual health. It is very important to consider breast feeding for children because breast milk has lots of antibodies and it is easily digested by the newborn increasing the chance of her or his survival. In the verse 233 of Baqarah, Holy Quran, it is stated: mothers should feed their children two years.^32^

(vi) Development of cloning and existence of the cloned people in the society can lead to complications arising from the failure to recognize and distinguish; such as failure to recognize students, distinguish criminal from innocent, or recognize wife and husband among similar people and it is obvious that such complications result in anarchy and legal difficulties, and (vii) Cloning human with exceptional physical strength or intelligence and benefiting from them in aggression and oppression of others can be another harmful effect that can provide the background for modern slavery and exploitation of human.^32^

*Bioethical Issues in Cloning*

Bioethics as one of the new branches of “applied normative ethics” is a new field of research which reviews and analyzes challenges caused by using innovations and technologies in bioscience and biomedicine, and also regulates the does and does not in this area in the interdisciplinary space systematically.^33^ Considering bioethics in cloning, it refers to different ethical issues especially from religious and secular points of views even human therapeutic and reproductive cloning are not presented commercially, but animals are currently cloned and the technique is used in livestock production. In therapeutic cloning, generate tissue generation takes place to treat patients who cannot obtain transplants,^34^ resulting to avoidance of the need for immunosuppressive drugs,^35 ^and to stave off aging effects.^36 ^In reproductive cloning, parents who cannot procreate are advised to have access to the cloning technology.^35^

The protest against therapeutic cloning is just on the use of embyronic stem cells, which is related to the abortion debate.^35^ Regarding reproductive cloning, there are concerns that cloning is not yet highly developed to confirm the safety of the technology, and could be prone to abuse and concerns about how cloned individuals could integrate with the society.^37^^-^^40^ In 2015, about 70 countries declared banning of human cloning.^41^

*Principles on Elimination of Damages in Cloning*

The first principle states that nobody has the right to damage others and has no moral justification. Elimination of damage; especially, next to the principle of equality and non-discrimination will have more importance regarding ethical and human right interpretations. Considering human cloning, it is believed that the only type of cloning that may eliminate these harmful effects can be therapeutic cloning. In other words, the principle of elimination of harm states that the researches on cloning should not harm other humans and or cloned individual. Although cloning may have advantages to human generation such as prevention from genetic disorders and diseases, it may also result in reproduction of humans with specific capabilities and cause the abuse of the cloned individuals by others and its producers as tools. In this way, the cloned individual may suffer from unwanted harms while he basically plays no roles in accepting or refusing the harms.^42^

*Principles of Usefulness of Cloning*

This principle is considered as the second fundamental principle in the bioethics and it is stated that the hidden assignments in this principle prevent imposing harms and losses on others and it is close to conservative views of legal documents and moves toward promotion of goodness; but therapeutic cloning is not opposed in this area. In fact, it can be said that this principle is along with principle of elimination of harm. In other words, the researches should not harm the cloned individual and other people but work on his and other’s favor. Of course, the answer to what advantages the cloning can have for the cloned individual is not clear because the human existence differs from doubtful identity and relative is not considered as special advantage for the individual. If the difference is due to a specific capability, it seems that the specific capability is reproduced more for the benefits of others than the cloned individual himself.^42^

*Human End-in-Itself in Cloning*

Based on this principle which is stated as the third principle, all humans end in themselves and they have a dignity as a human. Thus, we are not authorized to disregard individuals to the level of devices and even animals to satisfy our research objectives in the area of biotechnology.^14^ Based on Kant’s formula of end-in-itself, any actions that cause to use humanity as a mere means not as end-in-itself, are forbidden and immoral. There are various interpretations of humanity end-in-itself: not to do anything about a human without his knowledge; respect his freedom, will and independency; help his happiness; and respect others’ humanity. Thus, based on Kant’s formula of end-in-itself, any cloning operations which disrespect the humanity of humans as a mere means for other purposes are forbidden.^29^

Therefore, the cloning is forbidden to reproduce and replicate a large group of the cloned humans for the purposes of war or in peace time, such as: hard and overwhelming works, reproduction of useful humans for the society such as the genius of science, politics, and military, and to reproduce children of desired genotypes, and to replace newly-dead spouse, children or relative. In such cloning, humanity of the reproduced humans is not the purpose, but the developing of the society and the meeting of demands of other humans. It seems that the cloning to reproduce a child for infertile couple and the therapeutic cloning (providing that the beginning of humanity and human dignity is not considered from the time of fertilization and conception) to reproduce transplanted organs, is authorized because humanity is not a mere means.^29^

In therapeutic cloning, because the current technology is used for welfare, treatment and generally, for serving human and humanity, it is human who is the purpose and it does not conflict with Kant’s formula of humanity fundamentals; (Of course, if we do not consider the embryo as a human), because we solve the problem of some of humans and use some others as a mere means (because all humans do not need this technology). It can be stated that all humans are not used as a mere means, but it should be taken into accounts that Kant’s purpose of not dealing with human as a mere means is quantitative and qualitative. He emphasized on the fact that humanity is not quantitative and should not be acted as a tool. In addition, he forbid the use of human as a tool even by the person himself.^43^

The respect to human dignity is in a manner that it is highly considered in the international rules and declarations; for example, in the introduction and some of the articles of International Declaration on Human Genetic Data, 2003, observing the human dignity is a must and also the first article of International Declaration on the Human Genome and Human Rights, 11 November 1997, the human genome is considered as part of human heritage and it declares that human genome is the principle of fundamental unity of all members of the human family and the need for recognition of their inherent dignity and distinction and the article 11 of the declaration knows the human reproductive cloning in contrary to human dignity.^44^

*Principles of Reciprocity in Cloning*

Generally, this principle states that “Act others as you desire to be acted”. Kant’s formula of the universal law regards the same notion. In fact, the formula of human end-in-itself together with this principle can improve normative system of Kant’s ethics. The principle corroborates ban of experiments on human cloning supposing that the clone is considered as human, but it is not believed that other types of cloning is in contrary with this principle. Of course, it is noteworthy that the principle encounters a basic challenge in the area of cloning; because basically, the possibility of reciprocity between the cloned individual and the researcher who reproduces it, is negated; that is, they both are not on equal terms providing reciprocity for both, but the cloned individual unintentionally becomes the objective of the research and the outcome is his different presence in the world of existence.^42^

*Violating the Principle of Informed Consent in Cloning*

One of the main principles of bioethics is the principle of consent. The individual’s consent is one of the issues of cloning operation. The issue considers the consent of the cloned product; that is, whether the cloned individual is satisfied with the cloning operation and permits the unnatural creation method? Obviously, the answer to the question is unknown because the clone does not exist at the time of cloning operation and he cannot state anything on the matter and after birth, the operation is completed and finished. Perhaps, some say that in the natural process, the newborn does not play any role in his birth and creation. In reply, it should be asked how the issues related to the many physical damages and hidden and unknown mental risks in the method of abnormal birth of the cloned child compared with the method of natural birth can be justified?^45^

Therefore, pursuant to ethical principles and potential risks of cloning operations, further contemplations are needed on the technology and it should be avoided at least until its hidden aspects are clearly revealed. In regard to consent of the cloned child, the consent and permission of the donor of oocyte, the pregnant mother and even the donor of the somatic cell are also considered and it is an issue which can be harassed and abused.^45^

With a review on the mentioned principles, we conclude that the researches on reproductive cloning should include the following six features: (i) Be advantageous to society and impossible for any other methods, (ii) Previously operated on animals, (iii) Operated in a manner that all types of unnecessary physical and mental pains are prevented, (iv) If death or deficiency of the clone is probable, the operation is prevented, (v) Actions taken to protect the individual against damages, deficiency or death, and (vi) The experiments should be stopped if the responsible researcher believes at any phase of the research, continuation of the researches may result in damage, deficiency or death of the tested individual.^42^

*Bioethical Analysis of Therapeutic Cloning*

The subject is more complicated about the therapeutic cloning. By using this technology, it is possible to obtain tissues immunologically compatible with the recipient and it is considered as a definitive treatment for diseases such as Huntington’s, Parkinson’s, Multiple Sclerosis, Myocardial Infarction, etc. Millions of patients around the world benefit from such researches but on the other hand, making such researches requires reproduction and then destruction of the developing embryo. Is it possible to reproduce a human embryo for one’s purpose, but it should be remembered that such embryos are the initial point of life of all human being. It is not right to cut the string of life of the cell collection at the beginning of life for the purpose of medical research.^46^

Anyway, assessing the advantages and disadvantages of this technology is complicating and difficult because on one hand, it is a promise of the great probable advantage to the humanity and on the other hand, it causes several moral doubts and concerns at the level of society. What adds more complications to this subject is that: first, it is not certain that scientists achieve what they claim. Second, there might be other alternatives with the same advantages and without the mentioned ethical issues. Such alternatives have already been proposed such as using adult stem cells. The most concerns made by the opposition about litigation of therapeutic cloning are on two axes.^46^

The first issue is the destruction of the initial embryos which is considered as disrespect of the newly-reproduced human and the initial point of human life. Second, there is the fear that if the reproductive cloning is banned and the therapeutic cloning becomes free, whereas the initial procedures and techniques of both of the methods are similar, the freedom is abused in this regard and the embryos are developed for the purpose of human cloning. This concern is so serious that the American government strongly criticized to United Nations in a declaration on putting therapeutic cloning out of control and knew it a way to operate reproductive cloning.^46^

Because the research institutes which clone the human embryo are able to use it for any purpose; for example, transferring the human embryo to a hired uterus and reproducing it to a human fetus. In spite of all respect for the new life in the frame of human embryo, supporters of therapeutic cloning believe that human dignity and legal status of the six-day embryo is never equal to a mature human and therefore, the moral problems arising from damage of the embryo are fewer than what the opponents claim. They consider an average value for human embryos and believe that using the human embryo at the first stage of development is not objected if cloning is operated in the precise legal framework.^46^

Some others believe that embryo is a string of cells and it is worth as much as other cells in a body; thus, doing researches on ancestral cell and therapeutic cloning are the same as other cellular and molecular biology researches and they do not have any types of moral problems. On the other hand, it is noteworthy that the purpose of reproduction of embryos is not “their destruction” but it is to serve life of humans and progress of medical science. To prevent from long-term culture of embryo and future abuse from it, the experiments will be done on embryos of less than 14-day-old. At this stage, organs are not differentiated yet. Supporters agree with the laws which put therapeutic cloning operation in the certain framework and by controlling the process of cloning operation prevent from any abuse and violation from the related regulations. They believe that the benefits of therapeutic cloning are so many that the technology cannot be ignored due to ethical problems.^46^

*Jurisprudential Analysis of Reproductive and Therapeutic Cloning*

This subject is important because reproductive and therapeutic cloning is considered a new technology and is different and various related aspects should be recognized and studied and put into the legal content.^17^

## JURISPRUDENTIAL ANALYSIS OF REPRODUCTIVE CLONING

*Jurisprudence of Sunni Scholars*

They discussed cloning with reasons such as interfering in God’s will, corruption on earth, changing tradition, variety, creation and breaking Muslims’ believes, and they expressed their comments by issuing Fatwa, resolutions and declaration. In idea of the religious intellects consider the sanctity of the matter to be so obvious that the opportunity for criticism and discussion of the followers in this area is closed. Some of Sunni scholars exceptionally authorize it in some cases such as treatment of infertility, providing that the technology is guaranteed to be harmless. To keep sanctity of human cloning, some declare the doubtful speech that the cloning process changes the creation process by God and it is an act of evil and forbidden. This challenge is stated by some of the Sunni scholars. They refer to some of the verses and cited comments to emphasize on sanctity of changing creation. Accordingly, change of creation is the temptation of Devil and Devil also tempts to corruption, prostitution and sins; thus, changes in creation is prohibited.^47^

*Shia Jurists*

Generally, the views of Shia scholars on human cloning can be classified in four categories of (i) 

The total permit for human cloning: Some of jurists and scholars allow the cloning due to lack of specific documents and clear evidence on the sanctity of cloning and according to the principle of permissibility. For example, Ayatollah Sistani and Ayatollah Fazel Lankarani considered human cloning not to be problematic if is limited to reconstruction of tissue damages. Also, Ayatollah Moosavi Ardabili believes that there is no strict reason for sanctity of human cloning and this operation is permissible if is limited to reconstruction of tissue damages.^17^

(ii) Limited permission on human cloning: Based on available documents and according to the first principle in this case, some authorities have allowed human cloning but they believe that if it is widely operated, it would be problematic; such as, recognizing the cloned individuals from one another, therefore, they give authorization by case and they do not allow it at large scale. According to reports by Professor Hassan Javaheri, there is no problem on cloning happening in nature, but it is not legal to be undertaken at large scale. (iii) Secondary sanctity of human cloning: Some of the Shia Jurists believe that human cloning is not a problem in nature based on their arguments, but operating it in laboratories may lead to inevitable corruption such as intervention in natural system.^17^

Therefore, to prevent from such corruption, the human cloning is considered as the secondary prohibition. Ayatollahs Seyed Kazem Haery, Sheikh Javad Tabrizi, Seyed Sadegh Shirazi, Yoosef Sanei and Naser Makarem Shirazi supported this statement. Ayatollah Makarem Shirazi responded to an exception in this issue: “Based on religious rules, it is not naturally forbidden but with respect to its probable side effects that may lead to disorders in the human society and they are obvious for experts, its operation will would be problematic.^17^

Ayatollah Yoosef Sanei also stated that normalizing and legalizing the cloning in a manner that it is considered the same as having children by marriage, is absolutely not compatible with Islamic regulations and jurisprudence and it results in corruptions which must necessarily be avoided legally, socially, ethically and developmentally. He has declared that the prevention and punishment of its perpetrators and attempters is a must and a rational and religious assumption for all humans especially, legal and executive authorities and propagators. However, he allowed human cloning in rare cases and necessities when it is beneficial for human health and also the use of its scientific aspects; such as the cloning of organs for treatment purpose.^17^

(iv) Ultimate prohibition of human cloning: The owners of this attitude basically prohibit human cloning as a sanction action and consider it as the ultimately illegitimate. According to changes in creation and based on the principle of non-possession of body for human and therefore, the danger and necessity of permissibility in this regard showed the ultimate prohibition of human cloning. Despite the four categories and disagreements, most of the jurists banned human cloning. In other words, although according to the principle of presumption of innocence, initially, most of the Islamic intellects ruled on its natural permissibility and those who agreed and prescribed the cloning mentioned some of the applications and functions of this technology. 

But ultimately, a large number of the Muslim jurists considered it as the secondary prohibition despite its primary and natural permissibility. Considering the consequences of such abuse, potential and actual corruptions and due to the necessity of life protection and respect to human dignity and the reputation of “the principle of no harm”, they emphasized on the necessity of prohibition of prescribing the process until clearance of all aspects of the issue and safety against probable risks and enough assurance in this regard.^17^

*Jurisprudential Analysis of Therapeutic Cloning*

The significance of jurisprudential analysis of therapeutic cloning is due to the unique features of the technique which play a crucial and exclusive role in treatment of incurable and deadly diseases. Despite such a wonderful role, whose aspects reveal development of scientific researches everyday, it is required that the jurisprudence has comment on the mentioned problem, the problem which is referred as the loss of ethical dignity and human right on the embryo. When embryo is developed, three actions can be undertaken including, (i) To allow to be destroyed, (ii) To place it in uterus where it develops into a human similar to the donor of the cell in terms of growth, and (iii) T use it to obtain stem cells.^48^

The operation is the third stage of therapeutic cloning which is described later. It should be known that which one of the three stages of therapeutic cloning is permitted and which is not? Naturally, if only one of the stages is considered prohibited, it is not possible to give fatwa of permission for therapeutic cloning which includes all the stages. The permission of therapeutic cloning is subject to permissibility of all the stages. Now to review the three stages: The first stage is the use of the cell from human body which is automatically not objected. If there is a problem, it is in the next stages which are not related to this stage.^48^

In the second stage, the cell is processed and developed for the next stage when the cell is at 6 or 7-day of age. In this process, three actions should be done: (i) Enucleation of cell, (ii) Placing it into the enucleated oocyte, and (iii) Simulating the oocyte by chemical or electrical current to start cell division. This type of manipulation in this stage is not prohibitive itself. The only problem is that it might be banned as the point of prohibition. Of course, this initial point is the time when we know that if the operation begins and due to loss of control, the opportunity of abuse in the situation is available and the development of human embryo becomes inevitable.^48^

If human cloning, either as primary or secondary, is a prohibited operation, the operation as the starting point of prohibition will be prevented. But, the third stage is the extraction of the hidden cell mass in the embryo for culturing and obtaining stem cells. This problem caused a serious disagreement in Christianity and Islam in this stage. The problem is the extraction of cell mass which results in disappearing and killing of the fetus and its potential to become a human. To rule out therapeutic cloning, it should be reviewed in three aspects of (i) To review the judgment as “fetal homicide”, (ii) To review the judgment as “sanctity for destruction of the embryo regarding the development in the murder case in the view of the judge and not regarding the customary murder”, and (iii) To review the judgment as “a mere prohibition of embryo destruction”, not prohibited regarding the murder.^48^

Naturally, there is a difference between the three categories. The category of sanctity for murder is more severe than the sanctity for the second and third categories. In the third one, the most important rule can be performed easier and more; that is, based on this theory, it can be said that although embryo destruction is banned, whenever a human is suffering from a severe illness and sometimes leading to death, with respect to the more importance of the human life, the embryo is allowed to be destroyed to obtain the stem cell to treat the patient.^48^

*Legal Analysis of Reproductive and Therapeutic Cloning in Iran*

As cloning is not still very common and is in the stage of development and has not been tested after birth, the countries with cloning technology do not have a complete and codified law for it. Human cloning may legally cause problems, including the reproduced individual that will be completely similar to the genetic donor, even his fingerprints, and it is exclusive for everybody and considered as the major factor to arrest the offender. So the genetic owner can commit a crime and escape from law, and allocate his action to the cloned individual or vice versa. Thus, the rights and freedom of both of them will be withdrawn.^31^

In addition, the real culprit will not be identified and the rights of the accused person will be ignored. The cloned human does not have a father (because it is not from the male sperm) and a mother (because it is not by composition of gamete) and a sister and a brother and a relative, and it is grown in the uterus which is not of his mother but the surrogate mother. In brief, he is an individual with no relativity. If a virgin woman has a child by cloning of her sexual cell, is her pregnancy legitimate or not? And is the born baby her clone or sister or daughter? Who does the cloned individual inherits? If somebody kills the cloned individual, what are the rules for compensation or retribution? And who is responsible for alimony and custodianship of the cloned individual? There are some other legal problems too.^31 ^It should be taken into accounts that any anti-science law cause the scientists and researchers to emigrate to other territories and societies with less strict laws. One of the tens of reasons for brain drain is lack of right and proper laws to protect scientists and intellects.^49^

At the time of writing the review, it is unlikely that individuals or centers in the country, process the idea of the human cloning and perhaps, they have made arrangements and taken into action in this field. This idea and the probability of its occurrence have revealed the lawful Iranian responsibility more than before and showed the necessity of taking immediate action to fill this legal gap.^17 ^On the other hand, now when the researches in the field of cloning have started, it is not possible to revert to or ban or ignore them instead, the right action is to direct the researches on cloning and pass the required rules of law for this field.^42^

It seems that the prospects of every country about therapeutic cloning are dependent on the worth of human embryo in the legal system. The truth is that even in the countries where abortion is considered as the criminal act and punishable, and exceptionally, the mother’s life is in danger or the fetus is malformed or even the embryo is a result of adultery rape, abortion is predicted. Undoubtedly, therapeutic cloning which is the final solution for the treatment and health of human leads to the destruction of embryo and cannot be placed among any of the aforementioned exceptions because the cloned embryo is merely destroyed for other’s health and not its existence endangers other’s life. Certainly, in the countries where the value of embryonic and fetal is not considered equal to life or even health of the human and due to different reasons, abortion is not legally banned, the therapeutic cloning encounter less challenges.^15^

To clarify the criminal liabilities of physicians and law of human cases in genetic experiments and new therapeutic methods such as cloning, the Iranian criminal laws should be studied. Unfortunately, the law of Iran has not changed along with developments and progresses in medical sciences, and Iran is one of the countries where law has not passed about cloning. The only available regulations in our country codified by consultative committees are affiliated to the research institutes include two documents and despite that they are called by laws, the executive bylaws of ethical principles in researches of medical sciences and ethical guides of researches on gametes and embryos, they lack legal standards and sanction and as their titles suggest, they should be called ethics doctrine. Thus, only those cases of Islamic penal code approved in 2013 and law on method of donating embryos to infertile couples approved in 2003 are to be responsive to new challenges.^17^

 Therefore, if an individual or individuals engage in human cloning, in terms of the legal fundamentals and legal principles, it is not legally possible to prosecute them because with respect to constitution law principle of “legality of offenses and penalties”, it is not possible to consider an act as a crime without a legal element and no punishment is considered for it and the action or leaving of the action can be considered as a crime that a law is passed for it and the action and leaving of the action is considered as a crime by the law and the related punishment is determined.^45^

Some might argue that based on principle 167 constitution law states that the judge is bound to endeavor to find the ruling on every case in the law and if not found, according to Islamic sources or fatwa, would issue the ruling. They cannot refuse handling of the case and issuing the ruling under the pretext of silence or deficiency or brevity or conflict of laws. The rule of law can be derived if required, a legal action can be taken into account. In reply to such cases, it should be said that on one hand, the principle mostly includes the civil cases and if its content is accepted in criminal issues, case issues not phenomenon to this extent, would be with effective outcome. The prospects of scholars and views of Islamic jurists and different fatwas and often conflicting responses with uncertainty in dealing with various issues of this technology, are additional reasons to the inadequacy of the response.^45^

Although mostly after the emergence of the phenomena and the related challenges, legislators take actions to pass and approve laws with respect to requirements and structure and sources and development capacities of civil rights and future legal researches, if the time gap lengthens between the phenomena and provision of the necessary related law, it causes corruption and irreparable consequences; especially, on the critical and vital issues which have created great concerns about human rights and criminal laws for the human of the third millennium.^17^

If person or persons practice human cloning, what legal acts are there to deal with them? Are there any rules considered in the related laws to take legal actions against the operators and users of the technology? If yes, to what extent are they expressive and comprehensive and if not, what should be done against the practice and the perpetrators? If any person or persons practice human cloning, what legal actions are there against them? Some of the practical policies which should be done include public education, description of probable risks and disadvantages of human cloning and placing religious and ethical scholars next to researchers of cloning.^17^

Therefore, we can conclude that the right method is guiding and controlling the cloning technology and banning the technique is not always fruitful. Of course, it should be taken into accounts that all are possible if the religion orders human cloning in the view of jurisprudence and is considered as permission. In other words, although the religious order on human cloning can be an absolute permission based on the strong principle of permission, it is not unlikely that in the future, corruption is proved to be real for them, Jurists rule it as secondary sanctity and even as primary one. If it is proved, the phenomenon is considered as example of required affairs based on creation of ethical, social and medical disorders. Religious and ethical rulings cannot be permission for it, and it seems that it is a point that only one case can be a response to it and it needs nothing but time.

## CONFLICT OF INTEREST

The authors declare no conflict of interest.
